# Recent Advances and Opportunities in the Study of *Candida albicans* Polymicrobial Biofilms

**DOI:** 10.3389/fcimb.2022.836379

**Published:** 2022-02-18

**Authors:** Carolina H. Pohl

**Affiliations:** South African Research Chair Initiative (SARChI) Research Chair in Pathogenic Yeasts, Department of Microbiology and Biochemistry, University of the Free State, Bloemfontein, South Africa

**Keywords:** *Candida albicans*, polymicrobial biofilm, biofilm formation, antimicrobial resistance, treatment options

## Abstract

It is well known that the opportunistic pathogenic yeast, *Candida albicans*, can form polymicrobial biofilms with a variety of bacteria, both *in vitro* and *in vivo*, and that these polymicrobial biofilms can impact the course and management of disease. Although specific interactions are often described as either synergistic or antagonistic, this may be an oversimplification. Polymicrobial biofilms are complex two-way interacting communities, regulated by inter-domain (inter-kingdom) signaling and various molecular mechanisms. This review article will highlight advances over the last six years (2016-2021) regarding the unique biology of polymicrobial biofilms formed by *C. albicans* and bacteria, including regulation of their formation. In addition, some of the consequences of these interactions, such as the influence of co-existence on antimicrobial susceptibility and virulence, will be discussed. Since the aim of this knowledge is to inform possible alternative treatment options, recent studies on the discovery of novel anti-biofilm compounds will also be included. Throughout, an attempt will be made to identify ongoing challenges in this area.

## Introduction

The opportunistic pathogenic yeast, *Candida albicans* can form biofilms on biotic and abiotic surfaces including implanted medical devices (Tsui et al., 2016). These biofilms are also often polymicrobial in nature, containing other fungi (Rossoni et al., 2015; Pathirana et al., 2019), bacteria (Khan et al., 2021) and viruses (Mazaheritehrani et al., 2014; Ascione et al., 2017). The proximity of all these organisms within a biofilm allows for interaction *via* physical means as well as through secreted compounds, such as quorum sensing molecules, redox active compounds and bioactive lipids (Fourie and Pohl, 2019). In addition, the availability of and competition for nutrients play important roles in the interaction (Trejo-Hernández et al., 2014; Arzmi et al., 2016; Fourie et al., 2018), which is often described as synergistic, competitive or antagonistic (Trejo-Hernández et al., 2014; Delaney et al., 2018; Garcia et al., 2020), although this may be a simplistic view as it is becoming evident that the interaction is complex and bi-directional (Fourie and Pohl, 2019). The outcome of the interaction may also be influenced by various host factors, adding another layer of complexity (Fourie et al., 2016; Negrini et al., 2019; Van Dyck et al., 2021).

Polymicrobial interactions may influence the expression of virulence factors (Trejo-Hernández et al., 2014; Morse et al., 2019; Farrokhi et al., 2021) and these biofilms are often more resistant to antimicrobial drugs than their monomicrobial counterparts (Ascione et al., 2017; Orazi and O’Toole, 2019; Little et al., 2021). In addition, polymicrobial interactions may also influence the host immune response and the outcome of infection (Bergeron et al., 2017). Thus, understanding these interactions, including the molecular mechanisms of regulation, is important to manage *C. albicans*-containing polymicrobial biofilms (PMBs).

Since the study of PMBs has gained increasing interest, especially during the last five years, this review will focus on the recent advances in the study of *C. albicans* PMBs, including the regulation and consequences for antimicrobial susceptibility and virulence. Recent work regarding discovery of novel anti-biofilm treatment options will be highlighted.

## Advances in Models Used to Characterize Polymicrobial Biofilms

In order to study the interaction between *C. albicans* and bacteria, various biofilm models are used (Gabrilska and Rambaugh, 2015). The different models are important to consider as they may influence the formation and regulation of biofilms, as well as their consequences. This will also impact the comparability of results across the different platforms Stoffel et al., (2020). Common *in vitro* models used to study *C. albicans* PMBs include those traditionally used to study single species biofilms and these have recently been reviewed by Chevalier and colleagues (2018). An exciting advance in the development of *in vitro* models for PMBs, is the *nBio*Chip platform developed by Srinivasan and co-workers (2017). This platform consists of hundreds to thousands of identical nanobiofilms, encapsulated in hydrogel spots. The cells are inoculated into suitable media and combined with cellulose based hydrogels. The cultures are robotically printed onto glass sides using a microarray printer and biofilm allowed to form, which are then scanned using a microarray reader. PMBs of *C. albicans* and *Staphylococcus aureus* were formed, and the biofilm morphology characterized. It was found that the PMBs consisted of filamentous *C. albicans* with *S. aureus* microcolonies interspersed in between the hyphae. An advantage of this model is that the biofilms can withstand washing steps common to most assays This facilitates high throughput downstream applications, such as screening for antimicrobial resistance as well as novel antimicrobial drugs. Despite the recent advances, *in vitro* models most often utilize nutrient rich media or physico-chemical parameters that do not necessarily reflect conditions in the host. During the last five years many models that attempt to better approximate the conditions within a host niche have been developed and improved upon.

One of the host niches that is almost synonymous with biofilms is the oral cavity and several models for PMB formation have recently been developed to more closely approximate the conditions in the host. These models include models that better mimic the availability of nutrients (Montelongo-Jauregui et al., 2016), the oxygen levels present in root canals (Toral et al., 2017; Abusrewil et al., 2020) as well as the various bacteria associated with either caries or soft tissue infection *in vitro* (Abusrewil et al., 2020; Young et al., 2021). In addition, commercially available organotypic oral mucosal models have been used to study the interaction between *C. albicans* and bacteria (Krishnamoorthy et al., 2020). However, a disadvantage of commercially available mucosal models, identified by Morse and co-workers (2018), is their high purchase costs. These authors developed cost effective three-dimensional mucosal models to evaluate the impact of denture associated biofilm infections by mono- and polymicrobial *C. albicans* containing biofilms in terms of tissue damage and indicated the potential of these models to be further developed to closer mimic the host responses. In addition to dentures, other abiotic surfaces (such as titanium used for dental implants) are also relevant to the oral cavity. An *in vitro* model of *C. albicans/Streptococcus gordonii* dual-species biofilms on titanium discs Montelongo-Jauregui et al. (2018) has also been developed. All these models indicated the formation of robust PMBs and synergistic cross-kingdom interactions between *C. albicans* and oral bacteria, under conditions that may be present in the oral cavity.

Another context in which PMBs are important, is wound infections. Townsend and co-workers (2016) developed a three-dimensional wound infection model on complex hydrogel-based cellulose to mimic diabetic foot ulcer PMB infection by *C. albicans*, *Pseudomonas aeruginosa* and *S. aureus*. They found that this model allowed the formation of structurally complex biofilms, which had a greater impact on antimicrobial susceptibility, compared to conventional *in vitro* biofilms.

*C. albicans* and *P. aeruginosa* PMBs have also been studied extensively in the context of the cystic fibrosis lung [reviewed in (Fourie and Pohl, 2019)]. However, *in vitro*, these studies do not consider biofilm formation under flow conditions (Kasetty et al., 2021). Kasetty and co-workers (2021) examined the dynamics of polymicrobial interaction using microfluidic devices and found that, contrary to static *in vitro* biofilm models, the interaction was not antagonistic towards *C. albicans*, but resulted in increased filamentation of *C. albicans* and increased biofilm biomass accumulation by both species. The impact that *C. albicans* filamentation may have on the formation of PMBs, was demonstrated by Ruiz-Sorribas and colleagues (2021) in an *in vitro* model of PMBs, containing *C. albicans*, *S. aureus* and *Escherichia coli*. The research used two different RPMI-1640 based media to obtain different levels of *C. albicans* hyphal formation. Their results indicated that biofilms with more hyphae had larger biomass and resulted in increased expression bacterial virulence-associated genes. In biofilms rich in hyphae, *S. aureus* virulence-associated genes, such as those encoding haemolysin delta (*hld*), haemolysin alpha (*hla*) and clumping factor A (*clfA*), were upregulated. Clumping factor A has a role in bacterial adhesion and promotes *S. aureus* colonization of biotic and abiotic surfaces (Herman-Bausier et al., 2018). Similarly, the gene encoding poly-N-acetylglucosamine synthase (*icaA*), responsible for exopolysaccharide synthesis was upregulated in hyphae-rich biofilms compared to hyphae-poor biofilms. This may indicate an increase in EXM production with implications for PMB formation and antimicrobial resistance.

The remaining challenge is that the complex nature of PMBs and the influences exerted on them by various parameters in the different models, currently complicates the comparison of data regarding formation and consequences (e.g. susceptibility to antimicrobial compounds) of PMBs across different studies (Chevalier et al., 2018; Stoffel et al., 2020). An important aspect to take into consideration when designing experiments and comparing data from various sources is the effect of priority (i.e. the order in which microbes are inoculated to form biofilms) (Cheong et al., 2021), as this may drastically influence the structure (and potentially the gene expression) of a biofilm. An example is the recently published study where the interaction between *C. albicans* and two bacteria, *S. aureus* and *Citrobacter freundii*, was investigated. When *C. albicans* and *C. freundii* were co-inoculated (neutral priority) or when *C. albicans* was given priority, the yeast formed hyphal networks, containing very few yeast cells, with *C. freundii* attaching and colonizing the hyphae *via* mannose-binding lectins. In these biofilms, the proportional abundance of *C. albicans* increased. The opposite was observed when *C. freundii* had priority, with no hyphae formed, but yeast cell aggregates formed on a dense *C. freundii* biofilm and decreased proportional abundance of *C. albicans*. Similar results were seen regarding proportional abundance under the different scenarios for *C. albicans* and *S. aureus*. Interestingly, in polymicrobial interactions involving all three organisms, *C. freundii* out-competes *S. aureus* for binding sites on *C. albicans.*

**Figure 1:**
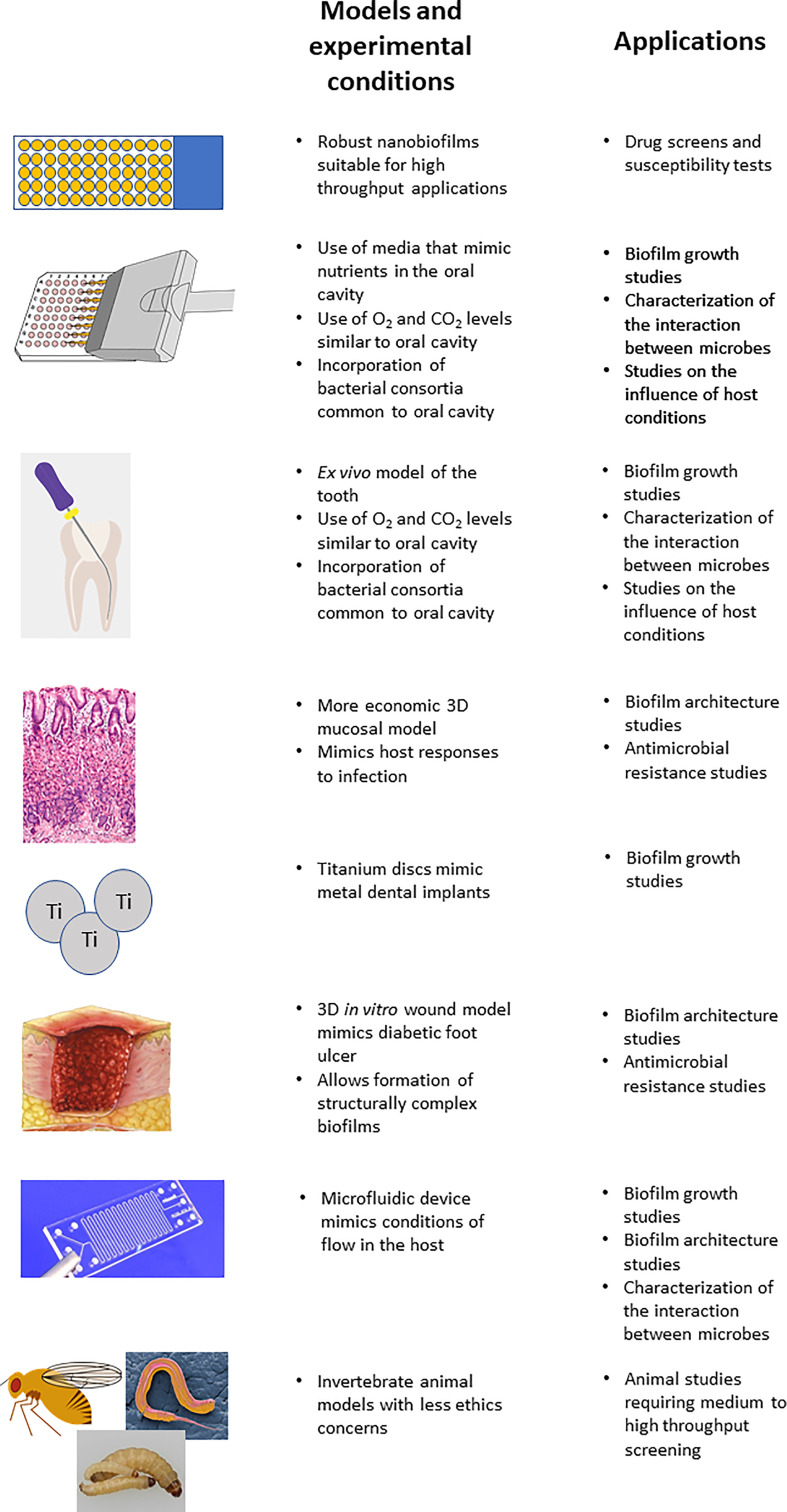
Summary of the models discussed in the study of polymicrobial biofilms.

In addition, although mammalian animal models are probably the most relevant to study *C. albicans* containing PMBs, it is impractical and unethical to use them in studies that require large numbers of animals, such as high throughput screening of potential anti-biofilm compounds. Here the use of alternative animal models, such as invertebrates (e.g. *Caenorhabditis elegans*, *Drosophila melanogaster* and *Galleria mellonella*) may provide a compromise (Gabrilska and Rambaugh, 2015; Holt et al., 2017; Farrokhi et al., 2021; Fourie et al., 2021; Mochochoko et al., 2021), and possible future endeavors could work to further establish them as PMB infection models. A summary of the PMB models, some advances made by them and possible applications are presented in **Figure 1**.

## Increased Understanding of the Complex Interactions

### Influence of Environmental and Host Factors

We have gained an increased appreciation of the complexity of the interaction between *C. albicans* and various bacteria, how the microbes influence each other and how the interactions may be influenced by the host as well as various environmental factors, including nutrient supply, oxygen levels, and the local microbiome (Krüger et al., 2019; Gaston et al., 2021). As expected, the different nutrients as well as their concentrations supplied by different media influenced biofilm architecture, biomass and metabolic activity (**Table 1**). Toral and co-workers (2017) determined the formation of *C. albicans*-*Enterococcus faecalis*, *C. albicans-S. aureus* biofilms as well as biofilms consisting of all three organisms on tooth surfaces under different atmospheric conditions, i.e. aerobic and microaerophilic (10% CO_2_). It was found that although there were differences in biofilm formation on the various parts of the tooth, generally the PMBs grew better in the microaerophilic atmosphere. Similarly, when the influence of different atmospheric condition (aerobic, 5% CO_2_, and anaerobic - 85% N_2_, 10% CO_2_, 5% H_2_) was evaluated on PMBs consisting of *C. albicans-S. gordonii-Fusobacterium nucleatum-Porphyromonas gingivalis* (Abusrewil et al., 2020), it was found that the greats biofilm formation occurred under 5% CO_2_ conditions. This may be due to the ability of both *S. gordonii* and *C. albicans* to grow well under this condition.

**Table 1:** Summary of the recent studies highlighting the impact of different media on *Candida albicans* containing polymicrobial biofilms.

Bacteria interacting with *C. albicans*	Media composition	Influence on polymicrobial biofilm	Reference
*Actinomyces naeslundii*	RMPI-1640 *vs* Artificial Saliva Medium	Biomass and metabolic activity were *C. albicans* strain and media specific	Arzmi et al. (2016)
*Streptococcus grodonii*	RPMI-1640, Todd Hewitt Broth + 0.02% Yeast Extract, 1:1 combination, synthetic saliva	Media composition influenced biofilm architecture, although all enabled synergistic interaction	Montelongo-Jauregui et al. (2016)
*Streptococcus mutans*	RMPI-1640 *vs* Artificial Saliva Medium	Biomass and metabolic activity were *C. albicans* strain and media specific	Arzmi et al. (2016); Brito et al. (2021)
	Different glucose concentrations	[Glucose] >60mM	
		↑ biofilm biomass	
		↑*C. albicans* CFUs/ml	
		↑ Insoluble EPS	

The presence of host factors such as heme may also influence the interaction in PMBs. Guo and co-workers (2020) found that competition for heme may not only influence *C. albicans-P. gingivalis* biofilm biomass, but that it may provide an advantage to the bacterium, increasing its vitality as well as resistance to serum and antibiotics. This advantage may be due to the ability of *P. gingivalis* to increase agglutination of erythrocytes and increasing its expression of heme utilization-related and gingipain expressing genes under low heme conditions. Host behavior, such as the use of nicotine, may also influence PMBs in the oral cavity. In a *C. albicans-S. mutans* PMB, nicotine caused a dose dependent increase in biofilm formation (CFU/ml as well as EXM production) by both organisms, although high levels of nicotine decreased the *C. albicans* CFU/ml (Liu et al., 2017).

*C. albicans* does not only interact with pathogenic bacteria, but also with commensal bacteria and other commensal fungi present in the host microbiome and serve to maintain microbial homeostasis (d’Enfert et al., 2021). Although several researchers have studied the impact of individual (or a few) members of the microbiome on *C. albicans* (as discussed in this review), the complexity of the microbiome poses challenges in gaining a more complete picture of how all the interactions within the microbiome. A recent example of the influence of a commensal bacterium on a PMB is found in the work of Huffines and Scoffield (2020) who found that the commensal bacterium, *Streptococcus parasanguinis*, influenced *C. albicans-S. mutans* PMB formation. In this context, *S. parasanguinis* decreased biofilm formation and restricted the incorporation of *C. albicans* into the PMB. IT also disrupted *S. mutans* glucose metabolism and caused a decrease in in glucosyltransferase activity and glucan synthesis.

### Regulation of the Complex Interaction

The mechanisms by which *C. albicans* and bacteria facilitate their interactions have also become clearer, and during the past decade some *C. albicans* genes were identified that play a role in these interactions. Dutton and co-workers (2016) found that deletion of *SAP9* (encoding a secreted aspartyl protease) caused *C. albicans* to form more compact biofilms and caused a decrease in incorporation of *S. gordonii, Streptococcus mutans*, *Streptococcus oralis*, *Streptococcus parasanguinis*, *Streptococcus sanguinis* and *Enterococcus faecalis* into dual species biofilms, suggesting that Sap9 may play a role in regulating the competition between *C. albicans* and oral bacteria. A possible role for *SAP9* as well as *ALS3* and *HWP1* in the interaction between *C. albicans* an *P. gingivalis* was also identified (Sztukowska et al., 2018; Bartnika et al., 2019). These genes were significantly upregulated in PMBs, grown under normoxic conditions. These authors further showed that the *C. albicans* adhesin, Als3, tightly interacts with the cell surface of *P. gingivalis* and that the bacterial gingipains and internalin (InlJ) are involved in this adhesion, although other proteins, such as enolase (Eno1) could also play a role. Als3, and possibly Als1, also mediate the interaction between *C. albicans* and *S. aureus* (Van Dyck et al., 2021). The interaction between *C. albicans* and *Actinomyces viscosus*, which is often associated with root caries, is considered synergistic, with greater biomass and EXM produced in these biofilms (Deng et al., 2019a). Deng and co-workers (2019b) identified that ergosterol may play a role in this specific interaction as *C. albicans erg11Δ/Δ* strains did not form enhanced biofilm biomass in PMBs. Another gene, involved in *C. albicans* biofilm formation and interaction with bacteria, is *SET3* (encoding a component of the Set3/Hos2 histone deacetylase complex) (Fourie et al., 2021). This gene was found to play a role during early *C. albicans-P. aeruginosa* biofilm formation as well as the virulence of mono- and polymicrobial infection in *C. elegans*, although the molecular mechanisms behind this regulation is still unknown.

Xu and co-workers (2017) identified one of the six known master regulators (Bcr1, Brg1, Efg1, Tec1, Ndt80 and Rob 1) of filamentation in *C. albicans*, Efg1, as important in the interaction between the yeast and *S. oralis*. *EFG1* was the only master regulator encoding gene that was upregulated in the presence of *S. oralis* in *in vitro* biofilms, organotypic oral mucosal models and on the tongue of orally infected mice. These authors also identified a downstream effector, Als1, (but not Als3 or Hwp1) that operated in concert with Efg1 in mediating the *C. albicans-S. oralis* interaction. Similarly, during the interaction between *C. albicans* and *S. gordonii*, mutants of three of the six master regulators (Brg1, Efg1 and Tec1), together with other regulators responsible for filamentation in *C. albicans* (Sfl1, Tup1 and Rim101) formed reduced biofilms with *S. gordonii* (Chinnici et al., 2019) and the authors speculated that this indicated that *C. albicans* filamentation was crucial for the development of robust PMBs. These authors also found that regulators involved in cell wall integrity and adhesion (Leu3, Cta4, Cas5 and Sko1) were negative regulators of *C. albicans*-*S. gordonii* biofilms. However, a study by Montelongo-Jauregui and co-workers (2019) challenged the idea that *C. albicans* hyphal formation and adherence are required for its interaction with *S. gordonii* in dual-species biofilms. They showed that *C. albicans* mutants (*efg1*Δ/Δ and *brg1*Δ/Δ), which are unable to filament and had biofilm formation defects, could form robust dual-species biofilms, with *S. gordonii* adhering to the yeast cells. These authors also showed that *S. gordonii* could form dual-species biofilms with an *als3*Δ/Δ mutant when the two were co-inoculated in synthetic saliva medium. This lack of Als3 requirement was confirmed using a *bcr1*Δ/Δ mutant, which does not express any of the Als adhesins, and point to the existence of other adhesive interactions that may be important in PMB formation. Interestingly, a study by Wan and co-workers (2021) found that the binding force between *C. albicans* and *S. gordonii* is approximately 2-fold higher than that between *C. albicans* and *S. mutans*. These interactions may also depend on the enhanced production of EXM by PMB (Montelongo-Jauregui et al., 2019) and specific nanostructures, such as *C. albicans*-produced nanofibrils (Veerapandian and Vediyappan, 2019).

Some of these surface interacting mechanisms that mediate the interaction between *C. albicans* and bacteria have been characterized. These include localized production of glucans that are responsible for the adherence of *S. mutans* to *C. albicans* hyphal cells in the oral cavity (Kim and Koo, 2020). *S. mutans* binding affinity to glucan coated *C. albicans* hyphae exceed the binding forces between *C. albicans* and *S. gordonii*, allowing for the formation of larger biofilm structures during early phases of biofilm formation (Wan et al., 2021). This may provide a mechanism for glucans to favor S. mutans binding interactions with *C. albicans* in the oral cavity. In addition to glucans, extracellular DNA (eDNA) found in the biofilm matrix also plays a role in the *S. mutans-C. albicans* interaction, especially during the initial attachment and early stages (Guo et al., 2021). The interaction between *C. albicans* and *Helicobacter pylori* was also further clarified, indicating strong attachment of the bacterial cells to the surface of *C. albicans via* multiple mechanisms (Palencia et al., 2022), including hydrophobic bonds between non-polar peptide chains or lipids on *C. albicans* cell walls and membranes of *H. pylori*. Hydrogen bonds may increase the strength of these interactions and formation of disulfide bonds between cysteine residues of surface proteins of both microorganisms may also be involved.

Naturally it is not only *C. albicans* genes and proteins that are responsible for the interaction between the yeast and various bacteria, as indicated by the involvement of *P. gingivalis* proteins highlighted above (Sztukowska et al., 2018; Bartnika et al., 2019). Karkowska-Kuleta and co-workers (2018) found that another of the extracellular virulence factors of *P. gingivalis*, a peptidylarginine deiminase enzyme, which converts protein arginine residues to citrullines, plays a role in the binding of *P. gingivalis* to *C. albicans*, possibly *via* citrullination of *C. albicans* surface proteins. In addition, during the initial interaction between *C. albicans* hyphae and *S. gordonii*, bacterial carbohydrate metabolism was seen to play a role as *fruR*, *fruB*, and *fruA* (encoding the transcriptional regulator, fructose-1-phosphate kinase, and fructose-specific permease) were consistently upregulated in the presence of *C. albicans* and deletion of these genes caused formation of less robust biofilms (Jesionowski et al., 2016). The streptococcal glucocyltransferase (Gtf), which synthesize α-glucan exopolymers, were also found to play a role in the interaction between *C. albicans* and *S. oralis* (Souza et al., 2020a). Interestingly, although deletion of *gftR* resulted in increased biofilm matrix, but not bacterial biomass, in monomicrobial biofilms, this deletion caused an increase in bacterial biomass in a PMB. The interaction between the commensal bacterium, *Streptococcus intermedius*, and *C. albicans* was found to cause increased biofilm formation (Mieher et al., 2021). Here *pas* (encoding a *S. intermedius* surface antigen, belonging to the Antigen I/II family of proteins) plays a role independent of Als3, as well as *srtA* (encoding sortase A – which allows for anchoring of surface proteins into the cell wall). Recently, Yang and co-workers (2018) also indicated the importance of the *S. mutans* adhesin, antigen I/II, for both the for the incorporation of *C. albicans* into the *C. albicans-S. mutans* biofilm and acid production by the biofilm. Interestingly, this interaction was also not dependent on the presence of Als1 and Als3. For all these studies, significant questions remain regarding the upstream signals and signaling pathways leading to the regulation of the various genes and mechanisms (Jakubovics, 2017).

## Consequences of the Complex Interaction

### Impact on Pathogenesis

**Figure 2:**
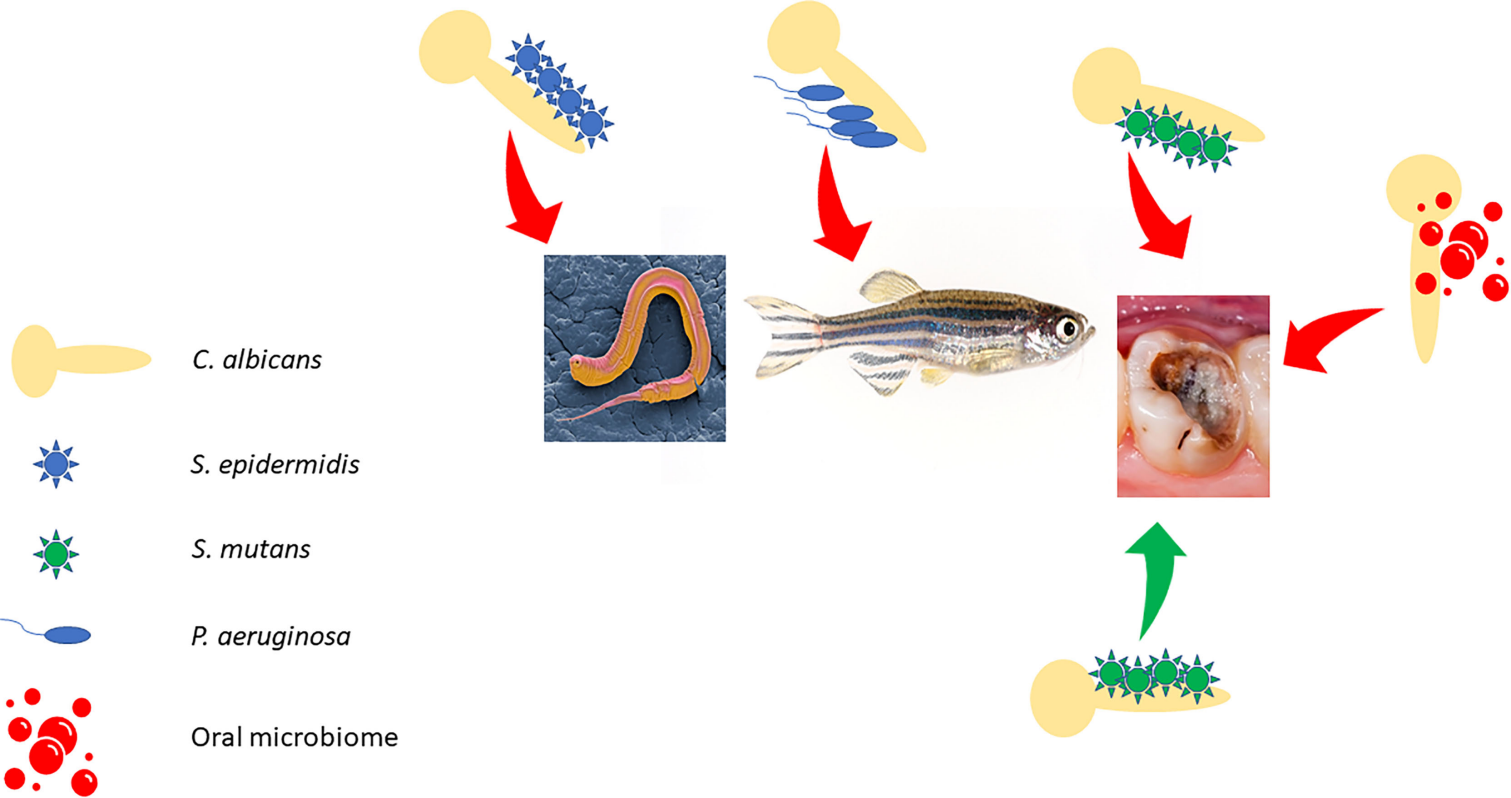
Outcome of polymicrobial biofilm infections in different hosts/host niches. Red arrows indicate increased pathogenicity, and the green arrow indicates decreased pathogenicity as discussed in the text.

The interaction between *C. albicans* and bacteria can influence the expression of specific virulence factors and may influence the outcome of co-infections (Allison et al., 2016; Dhamgaye et al., 2016) (**Figure 2**). Many of these co-infections are more pathogenic than single species infections. Examples include co-infection of *C. elegans* by *C. albicans* and *Staphylococcus epidermidis* (Holt et al., 2017) and co-infection of zebrafish by *C. albicans* and *P. aeruginosa* (Bergeron et al., 2017). This influence of *C. albicans* on pathogenesis in the oral cavity has received particular attention during the last six years. Sampaio and co-workers (2019) examined the ability of *C. albicans* to increase the cariogenicity of *S. mutans* (the bacterium involved in caries development) biofilms, using cultivation conditions that mimic the intake of sucrose during the day and lack of food during the night, over a period of 96h. They found that the PMB had greater volume and extracellular matrix (EXM) production and resulted in increased loss of dentine hardness.

Interestingly, the presence of *C. albicans* in caries-associated biofilms does not seem to always lead to increased cariogenicity. Willems and co-workers (2016) found that the interaction between *C. albicans* and *S. mutans*, may be less virulent. They grew PMBs for 24-72h on hydroxyapatite (HA) disks or glass slides and measured lactic acid concentrations, medium pH and the release of calcium from the HA disks. After 24h the medium pH of all biofilms decreased to below pH5.5, at which the enamel would be solubilized. However, after 72h, *C. albicans* was able to increase the pH to above pH5.5, even though more lactic acid was produced by *S. mutans*, possibly by metabolizing the lactic acid and producing ammonia, which increases the pH (Janus et al., 2017). This increase in pH caused a decrease in calcium release. Similar results were obtained by Eidt and co-workers (2019) who grew biofilms on enamel slabs in the presence of glucose or sucrose for up to 72h. Thus, the interaction with *C. albicans* can reduce the acidogenic and cariogenic potential of *S. mutans* biofilms. This interaction may be partially mediated by the *C. albicans* quorum sensing molecule, farnesol. At low concentrations, farnesol can reduce acid as well as EXM production by *S. mutans* biofilms (Fernandes et al., 2018).

These studies provide useful information on potential interactions between *C. albicans* and single bacterial species, but the *in vivo* applicability may still be limited as the oral microbiome is a complex community consisting of many microbes interacting with each other (Janus et al., 2017). Morse and co-workers (2019) increased our understanding of the impact of an oral bacterial consortium on the expression of virulence factors (i.e. hyphal formation and expression of virulence related genes). They found that PMBs consisting of *C. albicans* and a consortium of *S. sanguinis, S. gordonii, A. viscosus* and *Actinomyces odontolyticus* resulted in a significant increase in hyphal production. This increase was abrogated if *P. gingivalis* was also included in the consortium. In addition, a significant increase in the expression of *ALS3*, *EPA1*, *HWP1, PLD1*, *SAP4* and *SAP6* was observed in the PMBs without *P. gingivalis*. The addition of *P. gingivalis* to the consortium resulted in a decrease in the expression of all these genes except *SAP4*. *HWP1* and *SAP6* are associated with the hyphal phenotype, while *PLD1* is also required for hyphal formation under certain circumstances (http://www.candidagenome.org/) and their downregulation may correlate with the decrease in hyphal formation in the presence of *P. gingivalis*.

The impact of *C. albicans* on the oral microbiome was recently studied. Jauns and co-workers (2017) investigated this using saliva derived biofilms under various oxygen levels (anaerobic, aerobic, aerobic with 5% CO_2_). In anaerobic biofilms, no growth of *C. albicans* was observed, but in biofilms grown in the presence of oxygen, *C. albicans* did grow as part of the biofilm, forming extensive hyphae in the presence of CO_2_. Unexpectedly, *C. albicans* did not influence lactic acid production or protease activity in any of the biofilms. However, since hyphae can act as sites of interaction between *C. albicans* and bacteria, the authors investigated the influence of *C. albicans* on the microbiome of early biofilms in the presence of CO_2_. They found that more anaerobic bacteria (such as *Veillonella* spp., *Leptotrichia* spp., *Prevotella* spp. and *Fusobacterium* spp.) are present in *C. albicans* containing PMBs. These bacteria have been associated with various oral and dental infections. Similarly, Du and co-workers (2021) found that the introduction of *C. albicans* into biofilms obtained from saliva, resulted in significant changes in the microbial ecology, including increased growth of *Streptococcus* spp. and *Gemella sanguinis* as well as decreased growth of *Klebsiella pneumoniae*, *Haemophilus parainfluenza* and *Rothia dentocariosa*. However, in this study, the addition of *C. albicans* significantly increased acidogenicity and cariogenicity of these biofilms by upregulating bacterial genes associated with acid production. This increase in cariogenicity by *C. albicans* was also seen in a rat caries model. This interaction was dependent on *C. albicans PHR2*, which encodes a pH responsive glycosidase, indicating the importance of carbohydrate metabolism in this interaction.

These studies have shed light on the very complex interactions between *C. albicans* and bacteria and indicate that the species composition of a PMB can have a significant influence on the behavior of the biofilm members (including virulence). This would presumably hold true not only for oral biofilms, but for other biofilms both in the environment and the host.

### Impact on the Host Immune Response

The outcome of a PMB infection is not only dependent on the species composition of the biofilm, but also on the interaction between the biofilm and the host, especially the host’s immune system.

Cytokines are small proteins or glycoproteins that can influence both the innate and adaptive immune response. They are mainly produced by helper T-cells and macrophages, although cytokine production can be induced in almost all other cells. Many researchers have examined the influence of PMB infection on the production of cytokines by various cell types. The effect of mono-and polymicrobial biofilms of *C. albicans*, *S. mutans*, *S. gordonii* and *Aggregatibacter actinomycetemcomitans* on production of the cytokines, interleukin-8 (IL-8) and tumor necrosis factor-α (TNF-α), by whole blood was determined (Bhardwasj et al., 2020). They observed that both cytokines were most induced when blood was stimulated with the PMB as well as its supernatant, compared to any of the monomicrobial biofilms. However, supernatants of *C. albicans-P. gingivalis* biofilm reduced the production of IL-8 by monocyte-like cells although it did increase production of interleukin 1β (Bartnicka et al., 2020). The response by TNF-α was dependent on the incubation time since after an initial increase, the levels of this cytokine decreased. These authors found that the presence of *C. albicans* can stimulate *P. gingivalis* to produce gingipains, which can have proteolytic activity against cytokines, thus weakening the immune response. This protection from the host was confirmed in a mouse model of polymicrobial infection.

It is known that oral candidiasis may predispose a host to secondary systemic infection by *S. aureus* (Allison et al., 2019; Van Dyck et al., 2021). In this case adhesion of *S. aureus* to *C. albicans* as well the host immune response are needed for bacterial dissemination. It was found that macrophages co-cultured with *C. albicans* were highly attracted toward hyphae and preferentially engulfed *S. aureus* attached to the hyphae. This increase in phagocytosis provides more opportunity for dissemination of *S. aureus* to lymph nodes. Interestingly, although adhesion between *C. albicans* and *S. aureus* was not influenced by candidalysin [*C. albicans* secreted protein involved in immune response activation and immune cell recruitment (Naglik et al., 2019)], this protein was required for dissemination of *S. aureus* (Van Dyck et al., 2021). In addition, the interaction between PMBs on titanium and mucosal tissue was evaluated using a non-commercial *in vitro* organotypic mucosal model (Souza et al., 2020b). They found that *C. albicans*-*Streptococcus* spp. biofilms formed on titanium, caused increased tissue damage without increased proinflammatory cytokine production, compared to *C. albicans* monomicrobial biofilms.

These studies indicate the complex tri-partite interaction between *C. albicans*, bacteria and the host immune response that is still largely underexplored, although it will have significant impact on the outcome of co-infections and disease.

### Impact on Antimicrobial Resistance

It is well known that the close relationships between microbes may increase the resistance of one or more of the microbial partners to antimicrobials (Maisetta and Batoni, 2020). Several recent studies have increased our understanding regarding the influence of *C. albicans*-bacterial interaction on antimicrobial resistance of the microbes.

The *C. albicans-S. aureus* interaction has received significant attention in this regard, with various authors showing that the presence of *C. albicans* in the PMB significantly enhanced tolerance of *S. aureus* to vancomycin and that this is mediated by *C. albicans*-secreted molecules (Kong et al., 2016; Kong et al., 2017; Srinivasan et al., 2017; Vila et al., 2021). It was seen that β-1,3-glucan, a fungal cell wall component that is also found in the EXM, coats the bacterial cells, thus protecting them from the antibiotic (Kong et al., 2016). *C. albicans* also increased the tolerance of *P. aeruginosa* to the first line antibiotic, meropenem (Alam et al., 2020) as well as the tolerance of *S. gordonii* to clindamycin (Montelongo-Jauregui et al., 2019), possibly also due to the presence of *C. albicans*-secreted mannan and glucan. Another molecule secreted by *C. albicans* is the quorum sensing molecule, farnesol. Kong and colleagues (2017) found that this molecule increased oxidative stress in *S. aureus*, leading to upregulation of drug efflux pumps and subsequent increased vancomycin efflux.

Using a mouse subcutaneous catheter model of *C. albicans*-*S. aureus* biofilm infection, Vila and colleagues (2021) indicated that the observed *in vitro* vancomycin resistance was also observed *in vivo*. Further investigation revealed that *C. albicans* causes modulation of *S. aureus* biofilm related genes (including those that encode repressors of autolysis, lrg A and lrgB), which would lead to cell lysis and eDNA production. It was established that *C. albicans-S. aureus* biofilms do contain higher levels of eDNA and that DNAse treatment of these biofilms sensitized *S. aureus* to vancomycin and allowed the diffusion of vancomycin through the EXM. Therefore, it was proposed that in a mixed biofilm, the secreted/released matrix components and other molecules may protect *S. aureus* from this antibiotic. Interestingly, eDNA was also found to be involved in the increased miconazole resistance of *C. albicans* in *C. albicans-S. aureus* biofilms (Kean et al., 2017), indicating that the protective effect of PMBs are also applicable to *C. albicans*. This was further demonstrated by Kim and colleagues (2018), who saw that the *S. aureus* extracellular polysaccharide α-glucan, can directly bind to *C. albicans* cells and sequestered fluconazole, reducing uptake of the antifungal.

The clinical relevance of these studies was indicated by Hu and colleagues (2021) who observed that, in both local and systemic infection murine models, polymicrobial infection with *C. albicans* and *S. aureus* increased pathogenesis. In addition, *S. aureus* β-lactam and vancomycin resistance genes and *C. albicans* azole resistance genes were significantly upregulated in these infections, resulting in both antibiotic and antifungal drug resistant infections. It may also be speculated that the effect of these secreted compounds indicates that physical contact between *C. albicans* and bacteria is not a requirement for the observed increase in antimicrobial resistance.

In contrast, *C. albicans* matrix components and farnesol were found not to be involved in the increased resistance of *S. aureus* to other antibiotics (oxacillin, ciprofloxacin, delafloxacin, and rifampicin) (Nabb et al., 2019). Rather, PMBs were found to decrease the available glucose at a faster rate than mono-microbial biofilms. This enhanced metabolism was also observed by He and co-workers (2017) using RNASeq and biochemical analyses. According to Nabb and co-workers (2019), the competition for glucose results in decreased metabolism of *S. aureus*, with more cells containing less ATP and with lower membrane potential in polymicrobial cultures compared with monomicrobial cultures. These persister populations may explain the increased antibiotic tolerance. It will be important to see if this mechanism is also relevant *in vivo*, where glucose levels are often limited and greater competition for glucose, or co-utilization of alternative carbon sources, could be expected. The role of both bacterial persister cell in antibiotic resistance in the context of monomicrobial biofilms have been studied, however the role of *C. albicans* persister cells seem to be more controversial with different findings regarding the occurrence of persister cells in *C. albicans* biofilms (Denega et al., 2019; Galdiero et al., 2020b). However, the role of bacterial (or *C. albicans*) persister cells in polymicrobial biofilms, especially their influence on drug tolerance, has not been studied yet. Other *C. albicans*-bacteria interactions that influenced antimicrobial resistance, but for which the mechanism(s) have not yet been studied are indicated in **Table 2**.

**Table 2:** *Candida albicans*-bacteria interactions that influence antimicrobial resistance.

Bacteria	Antimicrobial	Outcome	Reference
*Cutibacterium acnes*	Micafungin	Increased *C. albicans resistance*	Bernard et al. (2018)
*Porphyromonas gingivalis*	Cefazolin	Decreased sensitivity of *P. gingivalis*	Guo et al. (2020)
	Sulfamethoxazole		
*Streptococcus gordonii*	Fluconazole	Increased resistance of polymicrobial biofilm	Montelongo-Jauregui et al. (2016)
	Amphotericin B	Increased *S. gordonii *resistance	Chinnici et al. (2019)
	Caspofungin		
	Clindamycin		
	Erythromycin		
	Ampicillin		
*Streptococcus mutans*	Chlorhexidine digluconate	Increased *C. albicans* and *S. mutans *resistance	Lobo et al. (2019)

Although the focus of most research regarding the consequence of PMB formation on resistance, has been directed towards antimicrobial resistance, this can be seen as a type of stress resistance and several recent papers have included other types of stress resistance, such as starvation resistance (Gao et al., 2016), resistance to non-antimicrobial drugs, such as nordihydroguaiaretic acid (Fourie et al., 2017), resistance to oxidative stress (Lobo et al., 2019) and serum (Guo et al., 2020). These studies all indicate that not only are PMBs generally more resistant to antimicrobials, but also to a variety of other stresses. Further mechanistic insights into this phenomenon may identify novel drugs or drug targets.

## In Search of Novel Therapeutic Solutions to Polymicrobial Biofilms

During the last five years numerous researchers have attempted to find novel treatment or prevention options for PMBs. Many of these studies have investigated plants or their extracts (**Table 3**).

**Table 3:** Plants and extracts with activity against *Candida albicans* containing polymicrobial biofilms.

Plant/extract	Antimicrobial used in combination	Bacterium interacting with *C. albicans*	Effect on polymicrobial biofilm	Reference
Commercial clove (*Syzygium aromaticum*) essential oil	Fluconazole	*S. aureus*	10x increase in anti-biofilm activity	Budzyńska et al. (2017)
	Mupirocin		4x increase in anti-biofilm activity	
Commercial rosemary (*Rosmarinnus officialis*) extract		*E. faecalis/*	Anti-biofilm activity	De Oliveira et al. (2017a)
		*P. aeruginosa/*		
		*S. aureus/*		
		*S. mutans*		
Commercial thyme (*Thymus vulgaris*) extract		*E. faecalis/*	Anti-biofilm activity	De Oliveira et al. (2017b)
		*P. aeruginosa/*		
		*S. aureus/*		
		*S. mutans*		
Cranberry extract		*S. mutans*	Inhibited cariogenic virulence properties	Philip et al. (2019)
Cajuputs candy (active ingredient is *Melaleuca cajuputi* essential oil)		*S. mutans*	Inhibited early biofilm development	Wijaya et al. (2019)
			↓ hyphal formation and bacterial adhesion	
Ethanol extract of Lerak (*Sapindus rarak*) seeds		Combination of *E. coli*, *P. aeruginosa* and *S. aureus*	Inhibits pre-formed biofilms and removes EXM	Pratiwi & Hamzah (2020)
Commercial lemongrass (*Cymbopogon flexuosus*) essential oil		*S. aureus*	Dose dependent anti-biofilm activity	Gao et al. (2020)
			Inhibition of EXM production	
Methanol extract of *Allium oschaninii*		*Klebsiella pneumoniae*	Inhibited biofilm formation	Galdiero et al. (2020a)
Methanol extract of *Allium ursinum*		*K. pneumoniae*	Inhibited biofilm formation	Galdiero et al. (2020a)
Ethanol extract of *Rhamnus prinoides*		*S. mutans*	Inhibited biofilm formation	Campbell et al. (2020)
LongZhang gargle (root and stem extract of *Toddalia asiatica* and *Cimicifuga foetida*		*S. mutans*	Inhibited biofilm formation	Gong et al. (2021)
			↓ hyphal formation, impacting biofilm structure	

Although the data from plant extracts seldom lead to treatment options in Western medicine, plants do contain several active compounds that, when purified, can inhibit growth of various microbes and may be assessed for their specific ability to inhibit PMBs (**Table 4**). Unfortunately, most of the research so far lack detail investigation of the mechanisms of action, or of toxicity studies in mammalian systems. Thus, making the translation of the *in vitro* results to application very limited.

**Table 4:** Plant-derived compounds with activity against polymicrobial biofilms.

Compound	Plant source	Bacterium interacting with *C. albicans*	Effect on polymicrobial biofilm	Reference
Curcumin	*Curcuma longa*	*S. mutans*	Inhibition	Li et al. (2019)
		*S. aureus-P. aeruginosa- E. coli*	Downregulation of *C. albicans* agglutinin-like genes	Tan et al. (2019)
			Inhibition	Ma et al. (2020)
				Hamzah et al. (2020a)
Gymnemic acid	*Gymnema sylvestre*	*S. gordonii*	Inhibit eDNA production, hyphal formation, adhesion. Downregulation of *S. gordonii gtfG1*)	Veerapandian and Vediyappan (2019)
Luteolin	Various	*E. faecalis*	Inhibition	Fu et al. (2021)
			Decrease in EXM production	
Nepodin	*Rumex crispus*	*S. aureus*	Inhibition	Lee et al. (2019)
		*A. baumannii*	Decrease hyphal formation	
Quercetin	Various	*S. aureus- P. aeruginosa- E. coli*	Anti-biofilm activity	Hamzah et al. (2020b)
Zerumbone	*Zingiber zerumbet*	*S. aureus*	Anti-biofilm activity	Shin and Esom (2019)
		*S. aureus-P. aeruginosa- E. coli*		Hamzah et al. (2020c)

Since biofilm formation is coordinated by quorum sensing systems, several researchers have targeted this system in the development of novel treatment options. The *C. albicans* quorum sensing molecule, farnesol, was found to reduce hyphal formation by *C. albicans* in a biofilm with *S. mutans* did not have any effect on the growth of the bacteria (Černáková et al., 2018; Rocha et al., 2018). Feldman and co-workers (2016) studied the antibiofilm activity of thiazolidinedione-8 and found that it was able to reduce biomass of *C. albicans-S. mutans* biofilms by affecting various processes, including S*. mutans* quorum sensing, EPS production and oxidative stress. The chemical could also affect *C. albicans* hyphal formation. This compound was successfully incorporated into a sustained-release membrane for possible application in clinical settings (Feldman et al., 2017). Phenazine-1-carboxamide, is a bacterial phenazine derivative – whose production is regulated by quorum sensing systems. This compound is well known for its antifungal activity (Peng et al., 2018) and was evaluated for its ability to inhibit *C. albicans*-*S. aureus* biofilms (Kanugala et al., 2019). It was found to completely inhibit biofilm formation when incorporated into porous silica nanoparticles and could successfully be used as antibiofilm coating on urinary catheters *in vitro*.

Other small molecules have also been studied for their effects on PMBs. Fluoride is known for its actions against cariogenic bacteria. Yassin and co-workers (2016) used this to develop fluoride releasing dental polymer that was able to inhibit the growth of *C. albicans-Lactobacillus casei-S. mutans* PMBs. Sodium trimetaphosphate is a supplement to fluoride in oral health and has beneficial effects on tooth enamel, but its effect on oral biofilms are not well studied (Cavazana et al., 2019), although it could reduce the metabolic activity and production of EXM by *C. albicans-S. mutans* biofilms. When combined with fluoride, it was also able to reduce biofilm biomass significantly. Another strategy was to test molecules with anti-*C. albicans* activity for their ability to inhibit PMBs. One such compound, 5-hydroxymethyl-2-furaldehyde, was found to inhibit *C. albicans-S. epidermidis* biofilm formation, by affecting attachment and virulence factors of *C. albicans* (i.e. hyphal formation, secretion of hydrolytic enzymes) as well as EXM, *in vitro* (Swetha et al., 2021). This compound also had a protective effect against infection in the *C. elegans* model. Another compound that has recently shown antibacterial and antifungal activity is auranofin – a trialkylphosphine gold complex. This compound was also recently shown to be able to inhibit *C. albicans-S. aureus* biofilms (She et al., 2020). Bay 11-7085 is another small molecule with possible antibacterial activity that could prevent formation of *C. albicans-S. aureus* biofilms, by inhibiting initial attachment and biofilm growth, especially of *C. albicans* (Escobar et al., 2021).

Various macromolecules, such as guanylated polymethacrylates, and branched polyethyleneimine based amphiphilic cationic macromolecules, were recently found to have activity against *C. albicans-S. aureus* biofilms (Qu et al., 2016; Mukherjee et al., 2020), but the most studied polymer was chitosan, with various authors indicating the ability of different forms/derivatives of molecule to inhibit PMBs (Tan et al., 2016; Ikono et al., 2019). This has led to the development of chitosan-coated surgical sutures and catheters that are able to prevent *C. albicans-S. epidermidis* biofilm formation (Prabha et al., 2021; Rubini et al., 2021). Chitosan has also been developed as a nanocarrier material for other potential antibiofilm compounds. Vieira and co-workers, (2019) used chitosan to coat iron oxide nanoparticles to form a carrier system for chlorhexidine. These nanoparticles showed similar or better results against *C. albicans-S. mutans* biofilms than free chlorhexidine. Chitosan nanoparticles loaded with curcumin was also effective against *C. albicans-S. aureus* biofilms (Ma et al., 2020). Similar nanoparticles were also loaded with cellobiose dehydrogenase and deoxyribonuclease I. These nanoparticles could penetrate the EXM to act against *C. albicans-S. aureus* biofilms and degrade eDNA in the matrix (Tan et al., 2020).

In recent years, the effectiveness of metal nanoparticles against *C. albicans* PMBs have also been studied. Silver nanoparticles were found to be effective against *C. albicans-E. coli* (Yasinta et al., 2021) and *C. albicans-S. aureus* biofilms and could be incorporated into silicone, that may be used for the manufacture of catheters (Lara and Lopez-Ribot, 2020) or maxillofacial prosthesis (Chong et al., 2021). Selenium nanoparticles (stabilized with either chitosan or bovine serum albumin) were tested for their ability to inhibit *C. albicans-S. aureus* biofilms (Filipović et al., 2021). The authors found that selenium nanoparticles stabilized with bovine serum albumin had reduced cytotoxicity towards mammalian cells, but significantly inhibited the dual species biofilms in a dose dependent manner. It was observed that *C. albicans* was more sensitive to these nanoparticles than *S. aureus* in the dual species biofilms.

Certain metal cross linked monomers have antibacterial activity and this was exploited to develop a dental pit and fissure self-adhesive sealant, containing di-n-butyl-dimethacrylate-tin, which exhibited good activity against *C. albicans*-*S. mutans* biofilms, without an effect on the mechanical properties of the sealant or causing cytotoxicity against mouse fibroblasts (Cocco et al., 2021).

Researchers have also focused on combination therapy in efforts to inhibit PMBs (**Table 5**). Although optimization of concentrations is often an issue in these studies (Rodrigues et al., 2017), this approach holds promise and clearly indicates the requirement for the addition of antifungal drugs when dealing with these types of biofilms. The importance of antifungals in the treatment of PMBs was also demonstrated by Luo and co-workers (2021), who found that *C. albicans-S. aureus* biofilms treated with amphotericin B, exhibited not only a reduction in *C. albicans* cell numbers, but also a decrease in bacterial cells – due to the supportive role of *C. albicans* hyphae in the development of these biofilms.

**Table 5:** Combination therapy against polymicrobial biofilms.

Drug combinations	Bacteria interacting with *C. albicans*	Effect on polymicrobial biofilm	Reference
Polymyxin and amphotericin B	*P. aeruginosa*	Combination of amphotericin B and highest concentration of polymyxin could eradicate polymicrobial biofilms	Rodrigues et al. (2017)
Flucloxacillin, ciprofloxacin and fluconazole	*S. aureus* and *P. aeruginosa*	Combination of all three drugs was required to inhibit all three microbes in the biofilm	Townsend et al. (2017)
Anidulafungin and tigecycline	*S. aureus*	Synergism against *in vivo* intra-abdominal biofilms	Rogiers et al. (2018)
2-aminobenzimidazole and curcumin	*S. aureus*	Increased biofilm inhibition by combination	Tan et al. (2019)
Caspofungin and polymyxin B	*P. aeruginosa*	Combination was able to significantly reduce the total biomass of mixed biofilms	Fernandes et al. (2020)
Antifungal chalcone-based derivative and antibacterial polycyclic anthracene-maleimide adduct	*S. aureus*	Synergistic biofilm inhibition	Bonvicini et al. (2021)
Berberine and amphotericin B	*S. aureus*	Synergistic biofilm inhibition with reduced hyphal formation and adhesion	Gao et al. (2021)
Farnesol combined with myricetin, C135* and compound 1771**	*S. mutans*	Eliminated bacteria from dual species biofilm	Lobo et al. (2021)
C135 and fluoride	*S. mutans*	Eliminated bacteria from dual species biofilm	Lobo et al. (2021)
Bacterial biosurfactant and DNase	*S. epidermidis*	Synergistic activity against biofilms	Srikanth et al. (2021)

*4-OH chalcone.

**5-phenyl-1,3,4-oxadiazol-2-yl)carbamoyl]methyl 2-{naphtho[2,1-b]furan-1-yl}acetate).

An alternative treatment option that has gained a lot of interest recently, is the use of antimicrobial peptides and peptoids. The amphiphilic nature of antimicrobial peptides allows them to bind to microbial membranes, causing disruption. A previously identified antifungal peptide product of the immunoglobulin gene, *IGHJ2*, was studied for its ability to inhibit formation of *C. albicans-P aeruginosa-S. aureus* PMBs (Di Fermo et al., 2021). This natural peptide was, however, not very effective in inhibiting biofilm formation *in vitro*. In a wound infection model, the peptide did cause a reduction in *S. aureus* and *C. albicans* numbers, but these were not statistically significant. The antimicrobial activity of natural peptides can be enhanced by various modifications to form synthetic peptides. Three such synthetic peptides have recently been demonstrated to be effective against *C. albicans-S. aureus* and *C. albicans-K. pneumoniae* biofilms *in vitro* and in mice and *G. mellonella* infections (Gupta et al., 2019; Galdiero et al., 2021a; Maione et al., 2021). Peptoids are peptide-mimics with increased resistance to proteases and therefore better stability *in vivo* (Luo et al., 2017). Three peptoids were tested against *C. albicans-S. aureus* and *C. albicans-E. coli* biofilms. Although these peptoids were able to reduce the cell numbers of *C. albicans* and bacteria, differences between the peptoids were observed depending on the composition of the dual species biofilms.

Other amphiphilic molecules that have been studied for their ability to prevent PMB formation, are biosurfactants, including lipopeptides, rhamnolipids and sophorolipids (Ceresa et al., 2021). Of the biosurfactants tested, one containing rhamnolipids was found to be most effective in preventing *C. albicans*–*S. aureus* and *C. albicans*–*S. epidermidis* biofilm formation. The antimicrobial properties of other lipids, including fatty acids, are well known and their effect on PMBs have also been evaluated. Saw palmetto oil, and the main fatty acids myristic acid (14:0) and lauric acid (12:0), were found to significantly inhibit *C. albicans-S. aureus*, *C. albicans-E. coli* biofilms as well as PMBs consisting of all three microbes *in vitro* and in *C. elegans* (Kim et al., 2021). Similar results were obtained for pentadecanoic acid (15:0) and pentadecanal against *C. albicans-K. pneumoniae* biofilms (Galdiero et al., 2021b). These lipids and lipid-based compounds may hold promise as a coating for medical implants (Ceresa et al., 2021; Galdiero et al., 2021b).

Although bacteriophages are an innovative strategy to combat polymicrobial bacterial biofilms (Mgomi et al., 2021), this is still unexplored in the context of *C. albicans* polymicrobial biofilms. Szafránski and co-workers (2017) did hypothesize how the interactions within the complex multispecies oral biofilm, containing *C. albicans* and various bacteria, may be targeted by bacteriophages directed at the different bacterial members. Interestingly, the *P. aeruginosa* phage, Pf4, was able to directly inhibit *C. albicans* biofilm formation as well as preformed biofilms. possibly by sequestrating iron (Nazik et al., 2017). These studies open the doors to further explore the applicability of bacteriophages in the inhibition of *C. albicans*-bacterial PMBs.PMBs can also form on non-implanted surfaces where disinfectants may be used to remove them. One such surface is dental unit waterlines. Costa and co-workers (2016) examine the ability of three recommended disinfectants to prevent or remove PMBs, consisting of *C. albicans, P. aeruginosa* and the amoeba, *Vermamoeba vermiformis*. The efficiency of the three disinfectants depended on their composition, and the concentration used, although none were able to completely eradicate mature PMBs. Of the three species in the biofilms, *C. albicans* was the most sensitive and *V. vermiformis* the least. Novel antiseptics, such as pyridoxine-based quaternary ammonium derivatives of terbinafine, are also investigated for their ability to eradicate *C. albicans-S. aureus* biofilms and was found to be very effective against both organisms (Garipov et al., 2020). The mechanisms of action include membrane damage and targeting of pyridoxal-dependent enzymes.

PMBs can also be inhibited without the use of chemicals. An example of such an approach is the use of atmospheric-pressure cold plasma, generated by gas ionization leading to the production of reactive oxygen and nitrogen species UV radiation and an electromagnetic field. This approach was able to inhibit both microbes in *C. albicans-S. aureus* biofilms *in vitro* and in reconstituted oral epithelium, without evidence of cytotoxicity (Aparecida Delben et al., 2016). Another nonchemical approach is the use of antimicrobial blue light (Ferrer-Espada et al., 2018). This antimicrobial action is thought to be mediated by intracellular porphyrins that act as photosensitizers. Exposure of a *C. albicans-P. aeruginosa* biofilm, grown in 96-well plates, to blue light at radiant exposure of either 216 or 500 J/cm^2^, caused a decrease in cell numbers for *C. albicans*, but an increased for *P. aeruginosa*, compared to their monomicrobial counterparts. Similar experiments performed using CDC biofilm reactor, showed a decrease in cells for both *C. albicans* and *P. aeruginosa* (Ferrer-Espada et al., 2019). It should however be noted that 500 J/cm^2^ caused significant reduction in metabolic activity of keratinocytes. Similar results were obtained by Tsutsumi-Arai and co-workers (2021), who indicated that both *C. albicans* and *S. mutans* cell numbers in a dual species biofilm are reduced by exposure to blue light, with *C. albicans* being more sensitive. The treatment caused an increase in reactive oxygen species, and this could be correlated to the higher levels of porphyrins present in *C. albicans* cells.

It is evident that significant research efforts are focused on finding novel therapeutic options for *C. albicans* containing biofilms, many of these studies included possible mechanisms of actions as well as cytotoxicity assays and *in vivo* testing. It is hoped that these practices will become more widespread and will increase the likelihood of finding options that have the potential to be translated from the laboratory (or field) to the clinical setting.

## Discussion: Future Prospects

We are entering an exciting time in the study of pathogenic microbes, where the intersection between traditional medical microbiology and microbial ecology is becoming more evident. This is to be expected since PMBs and polymicrobial infections form unique habitats where not only the microbes interact with each other, but also with the host, resulting in complex and often as yet unpredictable outcomes for disease progression and antimicrobial efficacy. It is envisioned that tools, such as metagenomic and metatranscriptomic analyses, more common place in microbial ecology may add significant new knowledge regarding not only the organisms present in polymicrobial biofilms in the host, but also the molecular interaction between them. This is indeed a complex field, but as indicated in this review, many exciting advances are being made.

## Author Contributions

The author confirms being the sole contributor of this work and has approved it for publication.

## Funding

Funding was provided by the National Research Foundation (NRF) of South Africa to CP (grant number 115566).

## Conflict of Interest

The author declares that the research was conducted in the absence of any commercial or financial relationships that could be construed as a potential conflict of interest.

## Publisher’s Note

All claims expressed in this article are solely those of the authors and do not necessarily represent those of their affiliated organizations, or those of the publisher, the editors and the reviewers. Any product that may be evaluated in this article, or claim that may be made by its manufacturer, is not guaranteed or endorsed by the publisher.
